# Are bacterial coinfections really rare in COVID-19 intensive care units?

**DOI:** 10.1186/s40001-023-01004-x

**Published:** 2023-01-21

**Authors:** Banu Karaca, Murat Aksun, Nagihan Altıncı Karahan, Senem Girgin, Bahar Ormen, Ahmet Salih Tuzen, Tuna Demirdal, Atilla Sencan

**Affiliations:** 1 Infectious Diseases Department, Faculty of Medicine, Atatürk Training and Research Hospital, Katip Celebi University, Basin Sitesi/Karabaglar, 35360 Izmir, Turkey; 2 Anesthesia and Reanimation Department, Faculty of Medicine, Katip Celebi University, Izmir, Turkey

**Keywords:** COVID-19, Bacterial coinfection, Intensive care unit

## Abstract

**Objectives:**

There are limited data about nosocomial coinfections of COVID-19 cases monitored in the intensive care unit. This study aims to investigate coinfections in COVID-19 patients followed in an intensive care unit of a university hospital.

**Methods:**

This study analyzed retrospectively the data of coinfections of 351 COVID-19 patients in the period 28.02.2020–15.01.2021 in a tertiary care intensive care unit in a university hospital.

**Results:**

Bacterial coinfections were present in 216 of the 351 cases. One hundred and thirty of these cases were evaluated as nosocomial infections. On the third day the Sequential Organ Failure Assessment Score, usage of invasive mechanical ventilation and presence of septic shock were significantly higher in the coinfected group. The neutrophil/lymphocyte ratio, polymorphonuclear leukocyte count, procalcitonin, ferritin, and blood urea nitrogen values were significantly higher in the coinfection group. White blood cells (WBC) (OR: 1.075, 95% CI 1.032–1.121, *p* = 0.001) and ICU hospitalization day (OR: 1.114, 95% CI 1.063–1.167, *p* < 0.001) were found to be independent risk factors for coinfection in the multivariate logistic regression analysis. The rates of hospitalization day on the day of arrival, the 21st day, as well as total mortality (*p* = 0.004), were significantly higher in the coinfected group.

**Conclusion:**

Bacterial coinfections of COVID-19 patients in the intensive care unit remain a problem. Identifying the infectious agent, classifying colonizations and infections, and using the proper treatment of antibiotics are of great importance in the case management of COVID-19 patients in the intensive care unit.

## Introduction

The ongoing COVID-19 pandemic caused by the SARS-CoV-2 virus is causing significant morbidity and mortality worldwide [1]. Intensive care monitoring is crucial because of severe pneumonia, acute respiratory distress syndrome (ARDS), and cytokine storms seen in the clinical course of the disease. In critical patients, bacterial and fungal coinfections can add to the clinical picture because of mechanical ventilation, the immune condition in the disease, and the predisposition caused by possible steroid use [2]. The SARS-CoV-2 infection causes damage primarily to B cells, T cells, and NK cells, causing a deterioration in the host’s immune system. Decreased lymphocyte count and impaired host immune response may cause COVID-19 coinfections [3]. Mortality related to high coinfection rates is higher in severe cases compared to moderate cases [4, 5]. In these severe cases, we could see secondary infections because of the use of invasive catheters and multidrug-resistant strains, such as *Acinetobacter baumannii, Escherichia coli, Pseudomonas aeruginosa*, and *Enterococcus spp*. Few studies have identified bacterial coinfections observed in COVID-19 cases monitored in intensive care due to extremely limited data. The incidence of nosocomial infections in COVID-19 intensive care units varies between %14 and 54 in different studies [2, 4, 5]. This study was planned to investigate bacterial coinfections in critical COVID-19 cases monitored in the intensive care unit.

## Methods

This study collected data from COVID-19 observed in the period 28.02.2020–15.01.2021 in a tertiary care intensive care unit (ICU) of a university hospital. There were 351 cases with COVID-19 lung involvement in the chest computed tomography (CT) scan and/or COVID-19-positive polymerase chain reaction (PCR) test. The data were retrospectively analyzed. The cases were monitored by anesthesiologists and infectious disease specialists for infection development. The specification and the classification of infections into community-acquired or nosocomial origins were made according to CDC and European Intensive Care Hospital Associated Surveillance Protocols [6–8]. The presence of sepsis was determined according to the definitions of Sepsis-3 [9]. Tracheal aspirate, phlegm, urine, wound site sample, and blood and catheter tip cultures were taken to determine the focus of the infection. A subgroup analysis was performed between the group of patients diagnosed with COVID-19 upon arrival or who were considered cases of coinfection by positive results after 48 h in the culture from clinical samples and the group that developed bacterial coinfection 48 h after hospitalization.

Patients were divided into the coinfection group (*n* = 216) and the non-coinfection group (*n* = 135). In addition to demographic data, comorbid diseases, SOFA (Sequential Organ Failure Assessment Score) scores on days 0 and 3, and mechanical ventilation usage were investigated. The correlation between the coinfection group and the non-coinfection group was examined in terms of infection sites, active microorganisms, antibiotic treatments, chest CT scans, laboratory findings, intensive care unit hospitalization times, septic shock development status, and mortality rates.

## Statistical analysis

Statistical tests were performed using SPSS version 19 (SPSS Inc., Chicago, IL, USA). Continuous variables are shown as mean value ± SD and categorical variables as the number of cases and percentage of the total number of patients. A student t-test or Mann–Whitney *U* test was used to compare parametric values between the two groups as appropriate. A Chi-square test was applied to compare categorical variables. Logistic regression analysis was used to identify independent predictors for coinfection. The factors entered into the multivariate model included those with *p*-values < 0.1 from the univariate analysis and variables with known predictive value. Also, spearman correlation analysis was performed to determine correlations among continuous variables and identify potential confounding factors. A two-sided *p* < 0.05 were considered statistically significant.

### Power analysis

The study needed to recruit 71 participants for each group to have 80.3% power with a 5% type 1 error level when assuming a coinfection rate of 45% in the ICU. The power of the study increased to 97.4% with the selection of 216 patients in the coinfection (+) group and 135 patients in the coinfection (−) group with a 5% type 1 error level.

## Results

Of the 351 patients, the mean age of the patients who developed coinfection was 66.0 ± 14.6 years, and the patients without coinfection were 63.6 ± 14.4 years. There were 84 (39%) female patients in the coinfection group and 50 (37%) in the non-coinfection group. There was no significant relationship in terms of the development of coinfection according to age and gender characteristics (*p* = 0.148, and *p* = 0.728). The most common comorbid diseases in the coinfection group were diabetes mellitus (DM) with 94 (61%) patients, hypertension (HT) with 118 (55%) patients, and coronary heart disease (CDH) with 57 (26%) patients. There was no statistically significant correlation between the coinfection group and the non-coinfection group regarding comorbid diseases. There was no statistical increase in coinfection in the postoperative cases monitored in the COVID-19 intensive care unit. The Sequential Organ Failure Assessment (SOFA) score, evaluated as a disease severity criterion, showed that, while there was no significant correlation between arrival SOFA scores (SOFA 0) and coinfection development, on day 3, SOFA scores were significantly higher in the coinfection group (*p* = 0.001). While the coinfection rate was not significant in the group using a high-flow nasal cannula (HFNC), a continuous positive airway pressure (CPAP) machine, or reservoir masks, it was significantly higher in cases with invasive mechanical ventilation usage (*p* < 0.005). Patients were monitored after 48 h of hospitalization in the ICU. Bloodstream infections and surgical site infections (SSI) were also monitored after 48 h of hospitalization, and infections that developed within 30 days after surgery were considered SSIs. In the coinfection group, the rate of community-acquired infections detected during hospitalization was 40%, while the nosocomial infection rate was 60% (Table 1). The most common causative microorganisms of community-acquired infections were *Staphylococcus aureus* and *Streptococcus pneumonia*. The subgroup analysis of the nosocomial coinfections indicated pulmonary infection in 85 (24%) cases, bloodstream infection in 48 (14%) cases, urinary tract infection (UTI) in 33 (9%) cases, and catheter-associated urinary tract infection (CAUTI) in 6 cases. The distribution of infectious agents was as follows: *Acinetobacter spp*. in 63cases, *Enterococcus spp.* in 24 cases, *Klebsiella pneumonia* in 16 cases, *Methicillin-resistant Staphylococcus aureus (MRSA)* in 9 cases, *Methicillin-resistant Staphylococcus epidermidis (MRSA)* in 13 cases, *E. coli* in 11cases, and *Pseudomonas aeruginosa* in 10 cases. The distribution of microorganisms according to the areas of infection is given in Table 2. The most common antibiotics used in nosocomial coinfected cases were piperacillin–tazobactam in 37 cases, meropenem in 78 cases, teicoplanin in 52 cases, colistin in 41 cases, and fosfomycin in 8 cases (Table 3). The mean duration of treatment was 11.4 ± 6.8 days in the coinfected group. The resistance patterns of the isolated microorganisms were as follows: carbapenem, cephalosporin resistance of gram-negative microorganisms, and methicillin resistance of staphylococci were 13.8%, 6.9%, and 17.1%, respectively. No significant correlation was found between the group with and without coinfection in terms of steroid usage, dose, and treatment duration.

**Table 1:** Demographic properties of the study population

Variable	Coinfection (−) (*n* = 135)	Coinfection (+) (*n* = 216)	*p*-value
Age^&^	63.6 ± 14.4	66.0 ± 14.6	0.148
Female, gender, *n* (%)	50 (37)	84 (39)	0.728
Comorbid diseases			
Diabetes mellitus *n* (%)	59 (39)	94 (61)	0.973
Hypertension *n* (%)	68 (50)	118 (55)	0.437
Chronic obstructive pulmonary disease n (%)	20 (15)	31 (14)	0.905
Coronary heart disease *n* (%)	38 (28)	57 (26)	0.718
Solid tm *n* (%)	7(5)	20 (9)	0.163
Hematological malignancies *n* (%)	2 (2)	7 (3)	0.310
Immunosuppression *n* (%)	4 (3)	4 (2)	0.497
Obesity *n* (%)	13 (10)	23 (11)	0.760
Chronic liver disease *n* (%)	2 (2)	5 (2)	0.587
Chronic kidney disease *n* (%)	10 (7)	22 (10)	0.379
Thyroid dysfunction *n* (%)	4 (3)	9 (4)	0.561
Dementia- Alzheimer *n* (%)	10 (7)	26 (12)	0.164
Postoperative status *n* (%)	3 (2)	12 (6)	0.133
SOFA* 0 day	7 (4–8)	7 (4–9)	0.092
SOFA* 3 day	7 (4–9)	8 (6–11)	0.001
MV n (%)	85 (63)	192 (89)	< 0.001
CPAP, *n* (%)	19 (14)	22 (10)	0.270
HFNC, *n* (%)	25 (19)	28 (13)	0.157
Reservoir O_2_ mask, *n* (%)	44 (33)	59 (27)	0.291
Simple O_2_ mask, *n* (%)	48 (36)	53 (25)	0.027
Nasal cannula, *n* (%)	7 (5)	9 (4)	0.656
ARDS at arrival, *n* (%)	114 (85)	186 (87)	0.402
Community-acquired infection n (%)	–	86 (40)	–
Nosocomial infection n (%)	–	130 (60)	–
Outcome Death 7 Day	27 (20)	40 (19)	0.731
Outcome 21 day	63 (47)	134 (62)	0.005
Hospitalization day*	13 (9–19)	16 (10–24)	0.014
ICU hospitalization day*	6 (4–11)	10 (6–14)	0.000
Mortality, *n* (%)	63 (47)	135(63)	0.004

*SOFA* Sequential Organ Failure Assessment, *MV* mechanical ventilation, *CPAP* continuous positive airway pressure, *HFCN* high-flow nasal oxygen, *O*_*2*_ oxygen, *ARDS* acute respiratory distress syndrome, *ICU* intensive care unit

Continuous variables are presented as mean ± standard deviation, and comparison was made using the student *t*-test at *p* < 0.05

Categorical variables are shown as number of subjects, with percentage of total number, and comparison was made using the Chi-square test at *p* < 0.05

^*^These variables are presented as median (interquartile range). Comparison was made using the Mann–Whitney *U* test at *p* < 0.05

**Table 2:** Infection agents according to coinfection regions in COVID-19 cases monitored in an intensive care unit

Infection type, agent	*n* (%)	Episode
Pulmonary	85	109
*Acinetobacter* spp.	52 (61)	
*E. coli*	6 (7)	
*Klebsiella* *pneumoniae*	11 (13)	
*Pseudomonas* spp.	9 (11)	
MRSA	7 (8)	
*Staphylococcus* epidermidis	1 (1)	
MRSE	4 (5)	
*Enterococcus* spp.	18 (21)	
*Proteus* spp.	1 (1)	
Blood	48	58
*Acinetobacter* spp.	22 (46)	
*E*. *coli*	4 (8)	
Klebsiella pneumoniae	6 (13)	
*Pseudomonas* spp.	5 (10)	
MRSA	6 (13)	
Staphylococcus epidermidis	4 (8)	
MRSE	6 (13)	
*Enterococcus* spp.	6 (13)	
Catheter	6	10
*Acinetobacter* spp.	4 (67)	
*E. coli*	2 (33)	
*Klebsiella* *pneumoniae*	1 (17)	
MRSA	28 (33)	
*Enterococcus* spp.	1 (17)	
Urinary	33	45
*Acinetobacter* spp.	11 (33)	
*E*.* coli*.	9 (27)	
*Klebsiella* *pneumoniae*	7 (21)	
*Pseudomonas* spp.	2 (6)	
MRSA	1 (3)	
*Staphylococcus* epidermidis	1 (3)	
MRSE	4 (12)	
*Enterococcus* spp.	9 (27)	
*Proteus* spp.	1 (3)	

E.coli; Escherichia coli, MRSA; *methicillin-resistant Staphylococcus Aureus, MRSE;* methicillin-resistant S. Epidermidis

**Table 3:** Antibiotics used in nosocomial coinfections

Antibiotics	*n* (%)
Piperacillin–tazobactam	37 (28)
Teicoplanin	52 (40)
Meropenem	78 (60)
Colistin	41 (32)
Fosfomycin	8 (6)
Antipseudomonal Cephalosporins	6 (5)
Tigecycline	5 (4)
Others	22 (17)

The examination of chest computed tomography (CT) findings showed that 21 (10%) of coinfected cases had less than 50% lung involvement, 108 cases had 50% or more involvement, and 82 cases had ARDS findings. Statistically, there was no significant difference between the coinfected and non-coinfected groups according to their radiological findings (*p* = 0.153). Patients diagnosed with septic shock were significantly higher in the coinfected group (Table 1, *p* < 0.001).

**Fig. 1:**
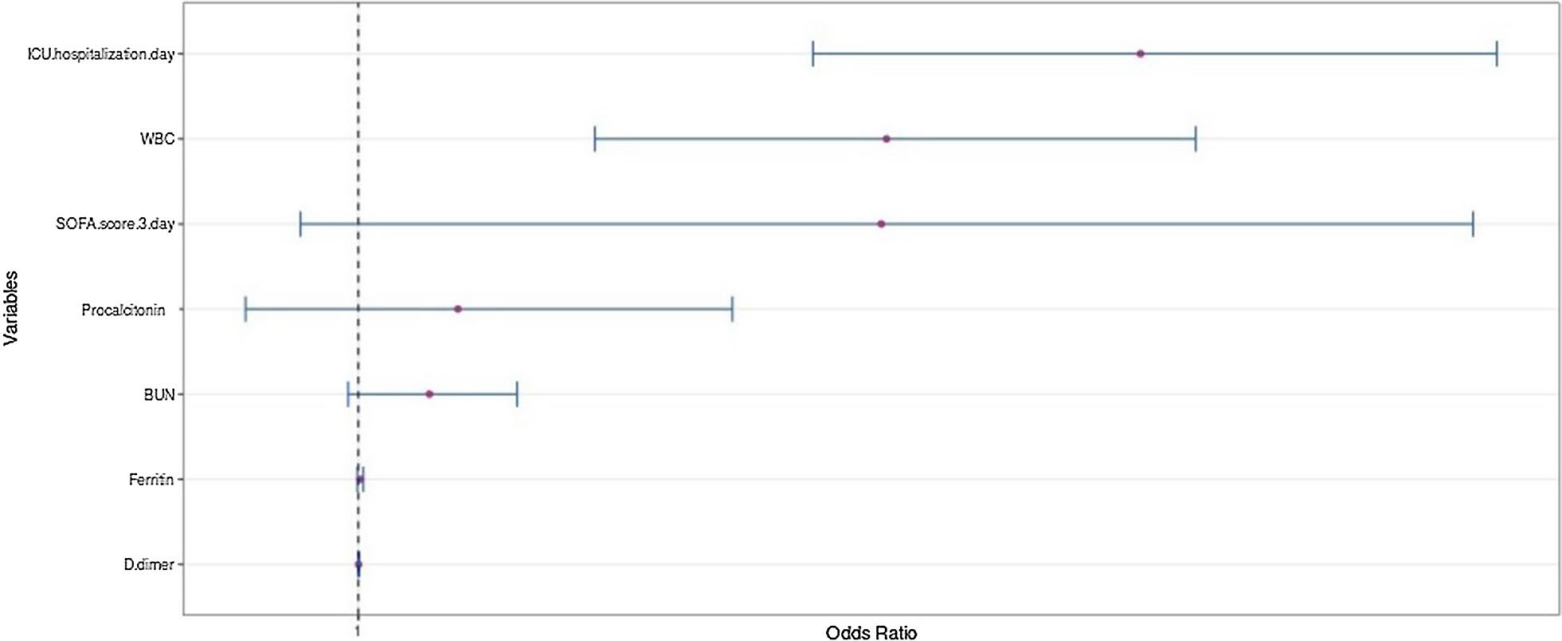
Multivariate logistic regression analysis with adjusted odds ratio for coinfection

According to laboratory data, neutrophil/lymphocyte ratio (*p* < 0.001), polymorphonuclear leukocyte (PNL) level (*p* < 0.001), procalcitonin positivity (*p* = 0.013), high ferritin levels (*p* = 0.002), and high BUN levels (blood urea nitrogen) (*p* < 0.001) were significantly correlated in the coinfected group (Table 4). White blood cells (WBC) (OR: 1.075, 95% CI 1.032–1.121, *p* = 0.001) and ICU hospitalization day (OR: 1.114, 95% CI 1.063–1.167, *p* < 0.001) were identified as independent risk factors for coinfection in the logistic regression analysis (Table 5 and Fig. 1). While no difference was detected between both groups when observing the mortality components on the 7th day, mortality on the 21st day was significantly higher in the coinfected group (*p* = 0.005). Total mortality was significantly higher in the coinfected group than in the non-coinfected group (Table 1, *p* = 0.004). The distribution of the mortality causes in the coinfected group was 40 cases from respiratory failure, 41 cases from septic shock, 50 cases from multiorgan failure, and 5 cases from sudden cardiac death (*p* = 0.003).

**Table 4:** Laboratory data of the study population

Variables	Coinfection (−) (*n* = 135)	Coinfection ( +) (*n* = 216)	*p*-value
White blood cell (× 10^3^/μL)^b^	9.6 (6.6–14.4)	13.7 (10.4–17.6)	< 0.001
Lymphocyte counts (× 10^3^/μL)^b^	0.74 (0.43–1.2)	0.7 (0.4–1.1)	0.422
Neutrophil/lymphocyte^b^	12 (5.2–19)	17.2 (9.4–27.1)	0.006
PNL, %^b^	8.1 (5.5–12.6)	12.3 (8.6–15.9)	< 0.001
Thrombocyte counts (× 10^3^/μL)^a^	263 ± 120	263 ± 113	0.955
Procalcitonin (µg/L)^b^	0.20 (0.09–0.76)	0.52 (0.16–3.28)	< 0.001
C-reactive protein (mg/dl)^b^	125 (83–175)	135 (82–210)	0.538
Blood urea nitrogen (mg/dl)^b^	23 815–34)	33 (21–52)	< 0.001
Creatinine (mg/dl)^b^	0.9 (0.7–1.2)	0.9 (0.7–1.6)	0.391
Aspartate aminotransferase (U/L)^b^	41 (25–74)	43 (27–79)	0.859
Alanine aminotransferase (U/L)^b^	31 (19–53)	35 (19–67)	0.403
D-dimer (µg/L)^b^	512 (330–1079)	993 (509–2778)	< 0.001
Ferritin (ng/ml)^b^	703 (338–1246)	954 (478–1650)	0.011
Fibrinogen (mg/dl)^b^	6.3 (5.1–8.2)	6.3 (4.3–8.2)	0.896
Troponin I (ng/mL)^b^	0.03 (0.01–0.10)	0.05 (0.02–0.10)	0.010
Lactate dehydrogenase (IU/L)^b^	407 (281–555)	429 (303–578)	0.692
POaO2/FIO2 (arrival)^b^	127 (98–156)	127 (87–180)	0.425
Lactate (entry) (mmol/L)^b^	1.5 (1–2)	1.6 (1.1–2.1)	0.239

PNL; polymorphonuclear leukocytes, FIO2; fraction of inspired oxygen

^a^Continuous variables are presented as mean ± standard deviation. Comparison was made using the student *t*-test at *p* < 0.05

^b^These variables are presented as median (interquartile range). Comparison was made using the Mann–Whitney *U* test at *p* < 0.05

**Table 5:** Univariate and multivariate analyses for predicting coinfection

Variable	Univariate			Multivariate		
	OR	95% CI	*p*-value	OR	95% CI	*p*-value
Simple O_2_ mask^b^	0.589	0.369–0.942	0.027			
MV^a^	4.706	2.716–8.153	< 0.001			
SOFA score 0 day ^b^	1.064	0.990–1.145	0.093			
SOFA score 3 day	1.116	1.043–1.194	0.001			
ICU hospitalization day	1.085	1.042–1.131	< 0.001	1.114	1.063–1.167	< 0.001
PNL^b^	1.102	1.057–1.149	< 0.001			
WBC	1.089	1.047–1.133	< 0.001	1.075	1.032–1.121	0.001
NLR^b^	1.039	1.019–1.060	< 0.001			
D-dimer	1.000	1.000–1.000	0.080			
Ferritin	1.000	1.000–1.001	0.002			
Procalcitonin	1.042	1.005–1.079	0.024			
Blood urea nitrogen	1.020	1.009–1.030	< 0.001			
Troponin I	0.977	0.864–1.105	0.710			

*OR* odds ratio, *CI* confidence interval, *O*_*2*_ oxygen, *SOFA* Sequential Organ Failure Assessment, *MV* mechanical ventilation, *ICU* intensive care unit, *WBC* white blood cell, *CRP* C-reactive protein*,* *NLR* neutrophil to lymphocyte ratio, *PNL* polymorphonuclear leukocytes

^a^As this parameter was included in SOFA score calculation, it was not entered into the multivariate analysis

^b^We conducted Spearman correlation analyses for continuous variables and selected one member of each pair of correlated variables (*r* > 0.3 and *p* < 0.05) to include in the logistic regression model to avoid multicollinearity

## Discussion

The presented study showed that bacterial coinfections developed frequently, especially in severe cases requiring intensive care monitoring for COVID-19.

Bacterial coinfections in viral pneumonia are especially common in patients in ICUs [10]. Primary infection or secondary bacterial pneumonia rates are 11–35% in patients infected with respiratory viruses [11]. This also applies to SARS-CoV-2 infection. Zhang and his colleagues reported a higher rate of bacterial coinfection (25%) in severe cases than in mild and moderate cases (0.8%) [12].

In this retrospective study, we screened COVID-19 cases with bacterial infections that developed within 48 h after attendance in a university hospital ICU. Infection was detected in 216 out of 351 cases, of which 130 (37%) were identified as nosocomial. This ratio is aligned with the literature [13]. Bardi et al. revealed that of the 140 critically ill patients with COVID-19 41% had bacterial or fungal infections on the 9th day of the intensive care unit. Also in a meta-analysis, including 30 studies and 3834 patients, 7% of hospitalized COVID-19 patients had a bacterial coinfection and this rate was higher in the COVID-19 intensive care unit [2]. In the analysis carried out in this group, the SOFA score was higher in the coinfected group on day 3. The SOFA score is a scoring system applied in intensive care patients with organ failure, and it has been reported in the literature that the disease is more serious in cases with a high SOFA score [14]. Bacterial coinfections increase the severity of the disease and speed up the progression to organ failure. Using HFNC, CPAP, and reservoir masks did not increase the development of coinfection. In addition, the coinfection rate was significantly higher in patients undergoing invasive mechanical ventilation. The increase in duration in the ICU increases and invasive treatments, such as mechanical ventilation, are applied, causing a risk for ventilator-related pneumonia [15]. These data are quite similar to the results of our study.

We found a statistically significant higher septic shock rate in the group with coinfection. It is a fact that bacterial factors have an important role in sepsis and septic shock, in this study, the incidence of septic shock diagnosis was found to be higher, in cases with a proven bacterial infection as expected.

In an evaluation of the infection sites, pulmonary infections were the most prevalent. This is compatible with the rate reported in the literature, which is in the range of 0–50% [16, 17]. However, one of the inclusion criteria in the study was a “positive CT scan.” These criteria increase the rate of pulmonary COVID-19 involvement and subsequently, possible coinfections. Therefore, the rate of pulmonary coinfection might be overestimated in the presented study. Blood, urine, and catheter culture positivity are the next most common site of infection in our study. The blood culture positivity rate is 3.8–33.5% in the literature [17]. Our blood culture positivity rate was similar to the literature. Some of these positivities suggest a positive result due to being contaminated with the skin flora and being taken under favorable conditions. Bardi et al. claimed that in the COVID-19 intensive care unit the most common infections were bloodstream infections (25%), pulmonary infections (23%), and urinary tract infections (8%) [13]. In our study, they reported a septic shock rate of 60% in the bacterial coinfected group and stated that bacterial coinfections were associated with a high SOFA score [13]. Congruent to our study, Humieres et al. and Baccolini et al. reported that pneumonia and subsequent bloodstream infection were the most common nosocomial coinfections [17, 18]. The distribution of infectious agents in nosocomial coinfections was as follows: *Acinetobacter spp.* in 63 (48%) cases, *Enterococcus spp.* in 24 (18%) cases, *Klebsiella pneumonia* in 16 (12%) cases, *Methicillin-resistant Staphylococcus aureus (MRSA)* in 9 (7%) cases, *Methicillin-resistant Staphylococcus epidermidis (MRSA)* in 13 (10%) cases, *E. coli* in 11 (8%) cases, and *Pseudomonas aeruginosa* in 10 (8%) cases.

In their 254-case series, which is compatible with our study, Baskaran et al. isolated 139 microorganisms from 83 patients, the most common of which were nosocomial pathogens, such as *Klebsiella pneumonia* and *Escherichia coli* [19]. In another study, *Pseudomonas aeruginosa* was the most commonly identified as a factor of nosocomial pneumonia [20]. As in our study, other studies have reported *Acinetobacter baumannii* as the most common nosocomial pathogen [18, 21]. Chen et al. reported that *Acinetobacter baumannii* and *Klebsiella pneumonia* were the most common bacteria that caused coinfection in 99 cases [22].

The duration of hospitalization in the intensive care unit and on the 21st day, as well as total mortality, were significantly higher in the coinfected group. The group with bacterial coinfection had a higher SOFA score on day 3, higher usage of invasive mechanical ventilation, prolonged hospitalization in the intensive care unit, higher incidence of septic shock, and a higher mortality rate on the 21st day. Similarly, mechanical ventilation and prolonged hospitalization duration in the intensive care unit were independent risk factors for coinfection [23]. The total mortality rate reported in the study by Bardi et al. in coinfected patients was similar to our study [13].

In severe COVID-19 cases, leukocyte count, neutrophil/lymphocyte ratio, procalcitonin, CRP, and ferritin elevation are reported in the literature [24, 25]. In our study using laboratory markers, the parameters that were detected as significantly higher in coinfected patients compared to the non-coinfected group were neutrophil/lymphocyte ratio, PNL, procalcitonin, BUN, and ferritin. When the literature was examined, Elabbadi et al. stated that they did not find any significant difference between the group with and without lymphopenia in terms of laboratory parameters in the COVID-19 cases followed up in the ICU [20].

In the COVID-19 pandemic, bacterial coinfections develop frequently, especially in severe cases requiring intensive care monitoring, increasing mortality drastically. There are insufficient data in the literature on these coinfections. Identifying the infectious agent, classifying colonizations and infections, and using the proper treatment of antibiotics are of great importance in case management. In addition, laboratory markers that may indicate infection should be considered in follow-up studies. Unnecessary antibiotic use should be avoided considering comorbid diseases, accompanying ARDS, and the multiorgan deficiencies of these cases. It is possible to reduce COVID-19-related mortality with appropriate and timely diagnosis and treatment.

## Data Availability

The data sets used during the current study are available from the corresponding author upon reasonable request.
